# Metagenomic biomarker discovery and explanation

**DOI:** 10.1186/gb-2011-12-6-r60

**Published:** 2011-06-24

**Authors:** Nicola Segata, Jacques Izard, Levi Waldron, Dirk Gevers, Larisa Miropolsky, Wendy S Garrett, Curtis Huttenhower

**Affiliations:** 1 Department of Biostatistics, 677 Huntington Avenue, Harvard School of Public Health, Boston, MA 02115, USA; 2 Department of Molecular Genetics, 245 First Street, The Forsyth Institute, Cambridge, MA 02142, USA; 3 Department of Oral Medicine, Infection and Immunity, 188 Longwood Ave, Harvard School of Dental Medicine, Boston, MA 02115, USA; 4 Microbial Sequencing Center, 7 Cambridge Center, The Broad Institute of MIT and Harvard, Cambridge, MA 02142, USA; 5 Department of Immunology and Infectious Diseases, 665 Huntington Avenue, Harvard School of Public Health, Boston, MA 02115, USA; 6 Department of Medicine, 75 Francis Street, Harvard Medical School, Boston, MA 02115, USA; 7 Department of Medical Oncology, 44 Binney Street, Dana-Farber Cancer Institute, MA 02215, USA

## Abstract

This study describes and validates a new method for metagenomic biomarker discovery by way of class comparison, tests of biological consistency and effect size estimation. This addresses the challenge of finding organisms, genes, or pathways that consistently explain the differences between two or more microbial communities, which is a central problem to the study of metagenomics. We extensively validate our method on several microbiomes and a convenient online interface for the method is provided at http://huttenhower.sph.harvard.edu/lefse/.

## Background

Biomarker discovery has proven to be one of the most broadly applicable and successful means of translating molecular and genomic data into clinical practice. Comparisons between healthy and diseased tissues have highlighted the importance of tasks such as class discovery (detecting novel subtypes of a disease) and class prediction (determining the subtype of a new sample) [1-4], and recent metagenomic assays have shown that human microbial communities can be used as biomarkers for host factors such as lifestyle [5-7] and disease [7-10]. As sequencing technology continues to develop and makes microbial biomarkers increasingly easily detected, this enables clinical diagnostic and microbiological applications through the comparison of microbial communities [11,12].

The human microbiome, consisting of the total microbial complement associated with human hosts, is an important emerging area for metagenomic biomarker discovery [13,14]. Changes in microbial abundances in the gut, oral cavity, and skin have been associated with disease states ranging from obesity [15-17] to psoriasis [18]. More generally, the metagenomic study of microbial communities is an effective approach for identifying the microorganisms or microbial metabolic characteristics of any uncultured sample [19,20]. Analyses of metagenomic data typically seek to identify the specific organisms, clades, operational taxonomic units, or pathways whose relative abundances differ between two or more groups of samples, and several features of microbial communities have been proposed as potential biomarkers for various disease states. For example, single pathogenic organisms can signal disease if present in a community [21,22], and increases and decreases in community complexity have been observed in bacterial vaginosis [23] and Crohn's disease [8]. Each of these different types of microbial biomarkers is correlated with disease phenotypes, but few bioinformatic methods exist to explain the class comparisons afforded by metagenomic data.

Identifying the most biologically informative features differentiating two or more phenotypes can be challenging in any genomics dataset, and this is particularly true for metagenomic biomarkers. Robust statistical tools are needed to ensure the reproducibility of conclusions drawn from metagenomic data, which is crucial for clinical application of the biological findings. Related challenges are associated with high-dimensional data regardless of the data type or experimental platform; the number of potential biomarkers, for example, is typically much higher than the number of samples [24-26]. Metagenomic analyses additionally present their own specific issues, including sequencing errors, chimeric reads [27,28], and complex underlying biology; many microbial communities have been found to show remarkably high inter-subject variability. For example, large differences are detected even among the gut microbiomes of twins [29], and both human microbiomes and environmental communities are thought to be characterized by the presence of a long tail of rare organisms [30-32]. Moreover, simply identifying potential biomarkers without elucidating their biological consistency and roles is only a precursor to understanding the underlying mechanisms of microbe-microbe or host-microbe interactions [33]. In many cases, it is necessary to explain not just how two biological samples differ, but why. This problem is referred to as class comparison: how can the differences between phenotypes such as tumor subtype or disease state be explained in terms of consistent biological pathways or molecular mechanisms?

A number of methods have been proposed for class discovery or comparison in metagenomic data. MEGAN [34] is a metagenomic analysis tool with recent additions for phylogenetic comparisons [35] and statistical analyses [36]. MEGAN, however, can only compare single pairs of metagenomes, as is also the case with STAMP [37], which does introduce a concept of 'biological relevance' in the form of confidence intervals. UniFrac [38] compares sets of metagenomes at a strictly taxonomic level using phylogenetic distance, while MG-RAST [39], ShotgunFunctionalizeR [40], mothur [41], and METAREP [42] all process metagenomic data using standard statistical tests (mainly *t*-tests with some modifications). Most methods for community analysis from an ecological perspective rely on unsupervised cluster analyses based on principal component analysis [43] or principal coordinate analysis [44]. These can successfully detect groups of related samples, but they fail to include prior knowledge of phenotypes or environmental conditions associated with the groups, and they generally do not identify the biological features responsible for group relationships. Metastats [45] is the only current method that explicitly couples statistical analysis (to assess whether metagenomes differ) with biomarker discovery (to detect features characterizing the differences) based on repeated *t *statistics and Fisher's tests on random permutations. However, none of these methods, even those offering nuanced analyses of metagenomic data, provide biological class explanations to establish statistical significance, biological consistency, and effect size estimation of predicted biomarkers.

In this work, we present the linear discriminant analysis (LDA) effect size (LEfSe) method to support high-dimensional class comparisons with a particular focus on metagenomic analyses. LEfSe determines the features (organisms, clades, operational taxonomic units, genes, or functions) most likely to explain differences between classes by coupling standard tests for statistical significance with additional tests encoding biological consistency and effect relevance. Class comparison methods typically predict biomarkers consisting of features that violate a null hypothesis of no difference between classes; we additionally detect the subset of features with abundance patterns compatible with an algorithmically encoded biological hypothesis and estimate the sizes of the significant variations. In particular, effect size provides an estimation of the magnitude of the observed phenomenon due to each characterizing feature and it is thus a valuable tool for ranking the relevance of different biological aspects and for addressing further investigations and analyses. The introduction of prior biological knowledge in the method contributes to constrain the analysis and thus to address the challenges traditionally connected with high-dimensional data mining. LEfSe thus aims to support biologists by suggesting biomarkers that explain most of the effect differentiating phenotypes of interest (two or more) in biomarker discovery comparative and hypothesis-driven investigations. The visualization of the discovered biomarkers on taxonomic trees provides an effective means for summarizing the results in a biologically meaningful way, as this both statistically and visually captures the hierarchical relationships inherent in 16S-based taxonomies/phylogenies or in ontologies of pathways and biomolecular functions.

We validated this approach using data from human microbiomes, a mouse model of ulcerative colitis, and environmental samples, in each case predicting groups of organisms or operational taxonomic units that concisely differentiate the classes being compared. We further evaluated LEfSe using synthetic data, observing that it achieves a substantially better false positive rate compared to standard statistical tests, at the price of a moderately increased false negative rate (that can be adjusted as needed by the user). An implementation of LEfSe including a convenient graphical interface incorporated in the Galaxy framework [46,47] is provided online at [48].

## Results and discussion

**Figure 1:**
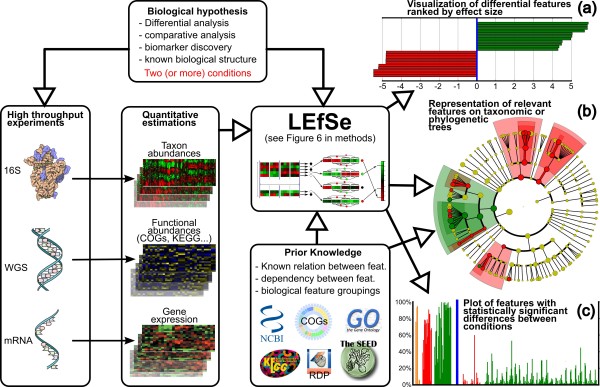
**LEfSe mines a wide range of high-throughput genetic data to find biologically relevant features characterizing one or more experimental conditions**. The inputs to the system are the specifications of the biological hypothesis under investigation (conditions and inter-condition sample groupings), the high-dimensional data obtained experimentally, and, optionally, prior knowledge from literature or databases used to define known relationships between features (used for meaningful hierarchical organization of the discovered biomarkers) or samples (used for testing biological consistency of potential biomarkers). LEfSe is a three-step algorithm (detailed in Figure 6). **(a) **LEfSe first provides the list of features that are differential among conditions of interest with statistical and biological significance, ranking them according to the effect size. **(b) **For problems with known hierarchical structure, either phylogenetic or functional, we then provide a mapping of the differences to taxonomic or functional trees. **(c) **Finally, the system produces a histogram visualizing the raw data within the specified problem structure for each relevant feature. While LEfSe has been developed primarily for metagenomic data containing taxon or gene abundances, it can be used for biomarker discovery in any setting where prior biological knowledge regarding the structure of a comparison is coupled with statistically significant differences in high-dimensional genomic features. KEGG, Kyoto Encyclopedia of Genes and Genomes; WGS, whole genome shotgun.

LEfSe is an algorithm for high-dimensional biomarker discovery and explanation that identifies genomic features (genes, pathways, or taxa) characterizing the differences between two or more biological conditions (or classes) (Figure 1). It emphasizes statistical significance, biological consistency and effect relevance, allowing researchers to identify differentially abundant features that are also consistent with biologically meaningful categories (subclasses; see Materials and methods). LEfSe first robustly identifies features that are statistically different among biological classes. It then performs additional tests to assess whether these differences are consistent with respect to expected biological behavior; for example, given some known population structure within a set of input samples, is a feature more abundant in all population subclasses or in just one? Specifically, we first use the non-parametric factorial Kruskal-Wallis (KW) sum-rank test [49] to detect features with significant differential abundance with respect to the class of interest; biological consistency is subsequently investigated using a set of pairwise tests among subclasses using the (unpaired) Wilcoxon rank-sum test [50,51]. As a last step, LEfSe uses LDA [52] to estimate the effect size of each differentially abundant feature and, if desired by the investigator, to perform dimension reduction.

We have specifically designed LEfSe for biomarker discovery in metagenomic data. We thus summarize our results here from applying the tool to 16S rRNA gene and whole genome shotgun datasets to detect bacterial organisms and functional characteristics differentially abundant between two or more microbial environments. These include body sites within human microbiomes (mucosal surfaces and aerobic/anaerobic environments), adult and infant microbiomes, inflammatory bowel disease status in a mouse model, bacterial and viral environmental communities, and synthetic data for quantitative computational evaluation.

### Taxa characterizing body sites within the human microbiome

Microbial community organization at multiple human body sites is an area of active current research, since both low- and high-throughput methods have shown both differences and overlaps among the microbiota of multiple body sites [53,54]. We examined these differences in the 16S-based phylometagenomic dataset from 24 individuals enrolled in the Human Microbiome Project [13,55]. A minimum of 5,000 16S rRNA gene sequences were obtained for 301 samples from 24 healthy subjects (12 male, 12 female) covering 18 body sites, including 6 main body site categories: the oral cavity (9 sub-sites sampled), the vagina (3 sub-sites sampled), the skin (2 sub-sites sampled), the retroauricular crease (2 sub-sites sampled), the nasal cavity (1 sample) and the gut (1 sample). We validated LEfSe by contrasting mucosal versus non-mucosal body site classes and by comparing three levels of aerobic environments (anaerobic, microaerobic, and aerobic). In both cases, the sub-sites within each class of body site were used as a biological subclass.

### Mucosal surfaces are colonized by diverse bacteria; non-mucosal microbiomes are strongly enriched for Actinobacteria

Our first analysis focused on differences in microbiota composition between mucosal and non-mucosal body sites. The oral cavity, gut, and vaginal sites were classified as sources of mucosal communities and the anterior fossa (skin), nasal cavity, and retroauricular crease as non-mucosal. Mucosal environments differ greatly from the other body sites, characterized primarily by interaction with the human immune system, oxidative challenge, and hydration [56].

**Figure 2:**
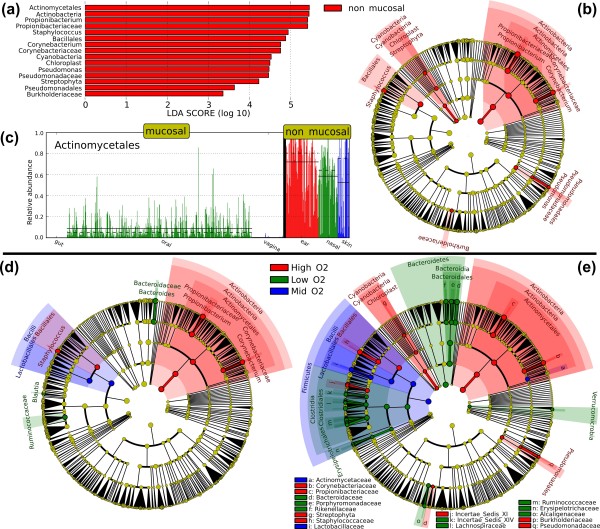
**LEfSe results on human microbiomes**. **(a-c) **Mucosal body site analysis. Mucosal microbial communities are diverse, while non-mucosal body sites are characterized by several clades, including the Actinobacteria. The analysis reported here is carried out on initial data from the Human Microbiome Project [55,56] assigning the main body sites to mucosal and non-mucosal classes, and using the body sites as subclasses. These graphical outputs were generated by the publicly available LEfSe visualization modules applied on the analysis results and integrating microbial taxonomic prior knowledge [58]. (a) Histogram of the LDA scores computed for features differentially abundant between mucosal and non-mucosal body sites. LEfSe scores can be interpreted as the degree of consistent difference in relative abundance between features in the two classes of analyzed microbial communities. The histogram thus identifies which clades among all those detected as statistically and biologically differential explain the greatest differences between communities. (b) Taxonomic representation of statistically and biologically consistent differences between mucosal and non-mucosal body sites. Differences are represented in the color of the most abundant class (red indicating non-mucosal, yellow non-significant). Each circle's diameter is proportional to the taxon's abundance. This representation, here employing the Ribosomal Database Project (RDP) taxonomy [58], simultaneously highlights high-level trends and specific genera - for example, multiple differentially abundant sibling taxa consistent with the variation of the parent clade. (c) Histogram of the Actinomycetales relative abundances (in the 0[1] interval) in mucosal and non-mucosal body sites. Subclasses (specific body sites) are differentially colored and the mean and median relative abundance of the Actinomycetales are indicated with solid and dashed lines, respectively. **(d,e) **Aerobiosis analysis. The cladograms report the taxa (highlighted by small circles and by shading) showing different abundance values (according to LEfSe) in the three O_2_-dependent classes as described in Results; for each taxon, the color denotes the class with higher median for both the small circles and the shading. (d) The strict (all classes differential) version of LEfSe detects 13 biomarkers whereas (e) the non-strict (at least one class differential) version of LEfSe detects 60 microbial biomarkers with abundance differential under aerobic, anaerobic, or microaerobic conditions. Additional file 2 reports the non-strict version of LEfSe focused on the Firmicutes phylum, highlighting several low-O_2 _specific genera within Ruminococcaceae and Lachnospiraceae.

LEfSe provides three main outputs (Figure 2), describing the effect sizes of differences observed among mucosal/non-mucosal communities, the phylogenetic distribution of these differences based on the Ribosomal Database Project (RDP) bacterial taxonomy [57], and the raw data driving these effects. LEfSe detected 15 bacterial clades showing statistically significant and biologically consistent differences in non-mucosal body sites (Figure 2a).

The most differentially abundant bacterial taxa in non-mucosal body sites belong to phyla with prevalent aerobic members: Actinobacteria, Firmicutes, and Proteobacteria, including environmental organisms from the Betaproteobacteria and Gammaproteobacteria clades. Non-mucosal overrepresented genera include *Propionibacterium*, *Staphylococcus *(found exclusively in non-mucosal samples), *Corynebacterium*, and *Pseudomonas*. Also of note is the relevant representation of plastids from plant organisms (chloroplasts), for which the distribution of associated taxa varies, as some are limited to non-mucosal surfaces (environmental exposure and potentially cosmetic products) and others to the digestive track (ingested food). No clades are consistently present in all mucosal body sites, demonstrating the β-diversity of these communities (that is, differences among their population structure), but many taxa within Actinobacteria, Bacillales, and several other clades are relatively abundant at all non-mucosal sites. The within-subject β-diversity at all phylogenetic levels is highlighted in Additional file 1, quantifying the extent to which distances among different mucosal body sites are larger than the equivalent distances among non-mucosal sites. This leads to a lack of taxa common to all mucosal body sites, and therefore no taxa are determined by LEfSe to be characteristic of the mucosa as a whole.

The Actinomycetales are usually the most abundant phylogenetic unit (order level) in non-mucosal communities, with percentages higher than 90% in several skin samples and at most 20% in the great majority of the oral mucosal samples and substantially lower in the vagina and gut (Figure 2c). From a quantitative viewpoint, the taxonomic order Actinomycetales makes up essentially all of the detected members of the phylum Actinobacteria, except in the vaginal site, which reported a substantial Bifidobacteriales presence. Bifidobacteriales themselves are not detected as differentially abundant between mucosal and non-mucosal body sites, since this is a feature only of the vaginal samples and not of all mucosal body sites. The contrast of many clades' abundance versus distribution is striking; for example, the genera *Alloscardovia*, *Parascardovia *and *Scardovia *are present in all body sites at very low abundances, while *Gardnerella *is overrepresented only in vaginal samples, with over three orders of magnitude difference in abundance. A similar commonality of distribution was found for the Bacillales at an even lower abundance. At the genus level, *Propionibacterium*, *Staphylococcus*, *Corynebacterium *and *Pseudomonas *are differentiated by both distribution and abundance. The *Staphylococcus *genus in particular is detected by LEfSe with a very high LDA score (more than five orders of magnitude), reflecting marked abundance in non-mucosal sites (mean 10%, 18% and 21% in the skin, retroauricular crease and anterior nares body sites, respectively) and consistently low abundance in mucosal sites (mean less than 0.001%).

### Classes with multiple levels: distinct aerobic, anaerobic, and microaerobic communities in the human microbiome

The roles of anaerobic metabolism in the commensal human microbiota have not yet been fully investigated due to the difficulty of studying these communities in culture. We thus further investigated the aerobicity characteristics of human microbial communities at a high level by grouping body sites into three classes with distinct levels of available molecular oxygen. The high-O_2 _exposure class includes body sites directly and permanently exposed to oxygen: skin, anterior nares and retroauricular crease. The mid-O_2 _exposure class includes the oral and vaginal body sites that can be directly, but not permanently, atmospherically exposed, and the low-O_2 _exposure class (the gut) is mainly anaerobic. The body sites included in the three classes may have other distinguishing features in addition to different oxygen exposure and, in general, these confounding factors can cause features unrelated with aerobiosis to be detected as biomarkers. However, the LEfSe biological consistency step assures that the detected biomarkers are characteristic of all the subclasses of a given class and with respect to all subclasses of the other classes. For example, the high-abundance of a bacterial clade in the mouth due to an oral-specific niche is not detected as a biomarker unless the same niche is also present in the vaginal samples (the other body site in the mid-O_2 _class) and not present in any high-O_2 _or low-O_2 _single body sites. So LEfSe will detect biomarkers more confidently connected with the aerobiosis characteristics than traditional methods that do not incorporate subclass information. Moreover, LEfSe is specifically able to analyze ordinal classes with multiple levels, and in agreement with established microbiology, we observed specific microbial clades ubiquitous within and characteristic to each of these three environments, detailed as follows (Figure 2d).

LEfSe allows ordinal classes with more than two levels to be analyzed in two different stringencies. The first requires significant taxa to differ between every pair of class values (that is, aerobicity in this example; see Materials and methods); the discovered biomarkers must accurately distinguish all individual classes (high-, mid-, and low-O_2_). In this example (Figure 2d; strict version), we detected 13 clades with LDA scores above 2, showing three distinct abundance levels. Alternatively, LEfSe can determine significant taxa differing in at least one (and possibly multiple) class value(s) (non-strict version); in other words, biomarkers that distinguish at least one individual class. Using this method (Figure 2e), we find 60 clades with LDA scores of at least 2.

Using either approach, each oxygen level is broadly characterized by a specific clade. The overall abundances of the Actinobacteria phylum are higher in body sites directly exposed to molecular oxygen with several members of the Actinomycetales order that colonize the skin. Actinomycetales includes the *Propionibacterium *genus, which is highly abundant on the skin, low in moderate-O_2 _environments, and absent from the gut. The Lactobacillales (primarily Bacilli) are specific to moderate O_2 _exposure levels, with conversely lower presences in the high-O_2 _exposure class, and are again absent from the gut. The Bacteroidaceae (particularly *Bacteroides*) are ubiquitous in anaerobic samples; interestingly, however, members of this family are more abundant in high oxygen availability conditions (particularly in skin and retroauricular crease) than in medium oxygen availability, showing the niche diversity within the phylogenetic branching. This is in agreement with observations that the microenvironment of many microbial consortia shows extreme biogeographical variation with respect to nutrients, metabolites, and oxygen availability [58,59].

### Bifidobacteria and additional clades are underrepresented in a mouse model of ulcerative colitis

Rodent models have been established to provide a uniquely accurate and tractable model for studying the gut microbiota, including the molecular and cellular mechanisms driving chronic intestinal inflammation [60-63]. In particular, mouse models of inflammatory bowel disease [63] facilitate a mechanistic evaluation of the contribution of the gut microbiota to the initiation and perpetuation of chronic intestinal inflammation, as occurs in human Crohn's disease and ulcerative colitis [64]. One host molecular mechanism known to maintain the balance between immune regulation and the commensal microflora is T-bet, a transcription factor expressed in many immune cell subsets. Its loss in the absence of an adaptive immune system results in a highly penetrant and aggressive form of ulcerative colitis [65] that is specifically dependent on and transmissible through the gut flora. We thus sought to investigate the characteristics of the fecal microbiota in a mouse model of spontaneous colitis that occurs in a colony of Balb/c *T-bet*^-/- ^× *Rag2*^-/- ^mice using 16S rRNA gene metagenomic data [66,67].

**Figure 3:**
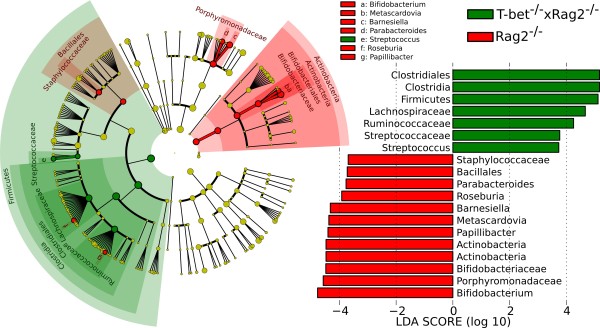
**Comparison between *Rag2*^-/- ^(control) and *T-bet*^-/- ^× *Rag2*^-/- ^(case) mice highlighting that, at the phylum level, Firmicutes are enriched in *T-bet*^-/- ^× *Rag2*^-/- ^mice, whereas Actinobacteria are enriched in *Rag2*^-/- ^mice**. In agreement with previous culture-based studies, *Bifidobacterium *species are underabundant in *T-bet*^-/- ^× *Rag2*^-/- ^mice [68], and LEfSe highlights several additional genus-level clades, including the specifically depleted *Roseburia *and *Papillibacter *within the otherwise overabundant Firmicutes.

LEfSe was applied to the microbiota data of 20 *T-bet*^-/- ^× *Rag2*^-/- ^(case) and 10 *Rag2*^-/- ^(control) mice (dataset provided in Additional File 10), finding 19 differentially abundant taxonomic clades (α = 0.01) with an LDA score higher than 2.0 (Figure 3). These differentially abundant clades were consonant with both our prior 16S rRNA-based sequence analysis using complete linkage hierarchical clustering and quantitative real time PCR-based experiments performed on the same fecal DNA samples [67]. More specifically, the marked loss in Bifidobacteriaceae and *Bifidobacterium *associated with *T-bet*^-/- ^× *Rag2*^-/- ^we observed here may explain the positive responsiveness of this colitis to a *Bifidobacterium animalis *subsp. *lactis *fermented milk product validated with low-throughput approaches [67].

At the family level, the *Rag2*^-/- ^enrichment of Bifidobacteriaceae, Porphyromonadaceae, Staphylococcaceae and the *T-bet*^-/- ^× *Rag2*^-/- ^enrichment of Lachnospiraceae confirm our reports in [68] using culture-based and quantitative real time PCR techniques. LEfSe's LDA score more informatively reorders these taxa relative to the *P*-values found for these families in our previous work, highlighting the Bifidobacteria and, interestingly, several clades within the Clostridia. These include the *Rag2*^-/-^-specific *Roseburia *and *Papillibacter *genera belonging to *T-bet*^-/- ^× *Rag2*^-/-^-specific families (Lachnospiraceae and Ruminococcaceae). The significant presence of *Metascardovia *(Bifidobacteriaceae) in *Rag2*^-/- ^mice is also interesting, as it may have a role similar to *Bifidobacterium *and because *Metascardovia *has been previously observed primarily in the oral cavity [68]. This analysis both highlights the agreement of LEfSe's effect size estimation with respect to low-throughput confirmations and suggests additional clades to be further investigated experimentally.

### A comparison with current metagenomic analysis tools using viral and microbial pathways from environmental data

We applied LEfSe to the environmental data of [69], a dataset with the goal of characterizing the functional roles of viromes (that is, viral metagenomes) versus microbiomes (that is, bacterial metagenomes). This task was used in [45] to characterize the Metastats algorithm on the same raw data. Among the 29 high-level functional roles (including unclassified roles) in the subsystem hierarchy of the SEED [70] and NMPDR [71] frameworks, LEfSe identifies only the 'Nucleosides and nucleotides' subsystem to be strictly differentially abundant among all environmental subclasses, specifically with higher levels in viromes than microbiomes. This is an accurate characterization of exactly the protein function most commonly encoded in viral genomes, whereas bacterial genomes of course encode a wide range of less specifically enriched functionality. When LEfSe is relaxed to detect significant variations consistent for at least one, rather than all, environmental subclasses, we additionally determine the 'Respiration' subsystem to be significantly enriched in microbiomes with respect to viromes, likely reflecting the uniformly aerobic bacterial metabolism captured by these data.

**Figure 4:**
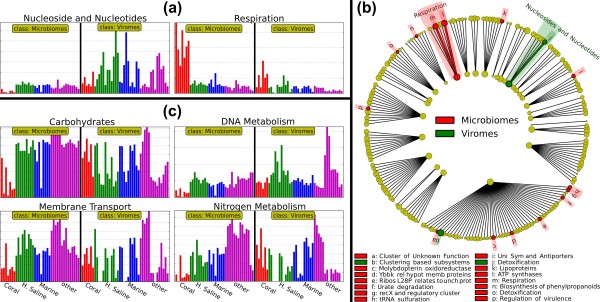
**LEfSe highlights pathways consistently differential between bacterial microbiomes and viromes within diverse environmental subclasses**. **(a) **Using the SEED [71] catalog of functional pathways, LEfSe reports Nucleoside and nucleotide metabolism and Respiration to differ consistently between bacterial microbiomes and viromes across environmental samples described in [70]. The former is significant using the strictest all-subclasses test, the latter in the more lenient one-subclass test. **(b) **A two-level cladogram reporting the significant pathway differences as visualized using the SEED hierarchy (see Additional file 3 for the three-level cladogram and detailed differences). **(c) **Metastats [45] reports four additional pathways differential among these data (Carbohydrates, DNA metabolism, Membrane transport and Nitrogen metabolism). Using only the KW test portion of LEfSe (α = 0.05), we obtain results consonant with Metastats (excluding Nitrogen metabolism). However, as shown here, an overview of the abundance histograms of these subsystems demonstrates them to be less consistent across environments (for example, Coral and Hyper-saline subclasses in the Carbohydrates, Membrane transport and Nitrogen metabolism) and to lose significance within individual subclasses (as for the DNA metabolism subsystem).

In addition to the Nucleosides and nucleotides and Respiration subsystems, Metastats [45] reports five other high-level functional roles as differentially abundant (*P *= 0.001). However, when taking the subclass structure into account across the sampled environments, these additional differences show much less consistent variation. This is demonstrated in Figure 4, which reports histograms of raw data for these cases and the different results of LEfSe, Metastats and the KW test alone. Moreover, since the subsystem framework is hierarchical (three levels), LEfSe's results include a cladogram showing the significant differences on each level (see Figure 4 for a two-level cladogram, and Additional file 2 for a three-level cladogram).

Considering all three levels of SEED functional specificity, LEfSe reports 59 subsystems to be more abundant in microbial metagenomes and only 7 that are more abundant in viral metagenomes (Additional file 3). Bacterial genomes encode a much greater quantity and diversity of biomolecular functionality than most viral genomes, and these differences are thus to be expected. However, they also highlight a consideration specific to most metagenomic (and, more generally, ecological) analyses, which typically analyze relative abundances. A few very common subsystems in viromes (that is, Nucleosides and nucleotides) will force the relative abundance of all other subsystems to decrease, resulting in apparent under-abundance. The subsystems detected to be virus-specific may thus show this trend in part due to the normalization of abundances in each sample. This issue is specific to neither LEfSe nor Metastats, however, and must be taken into account during interpretation of any relative abundance data, metagenomic or otherwise [72].

### Functional activity within the infant and adult microbiota indicates post-weaning microbial specialization

Just as LEfSe can determine whether organisms or pathways are differentially abundant among several metagenomic samples, it can also focus on individual enzymes or orthologous groups. Kurokawa *et al*. [73] analyzed 13 gut metagenomes from nine adults and four unweaned infants in terms of the functions of orthologous gene families. They originally did this by comparing the COGs [74,75] found in each metagenome to a reference database; later, White *et al*. [45] applied the Metastats algorithm to directly detect differences between infant and adult microbiomes. Using significance α values of 0.01 due to the low cardinality of the classes (in particular the infant class), LEfSe detected 366 COGs to be enriched in either adult or infant metagenomes, 17 of which have a LDA score higher than 3 (Additional file 4).

Among the 17 COG profiles with LEfSe scores higher than 3, 11 are also detected by Metastats. The six COGs not detected by Metastats (Additional file 5) are Outer membrane protein (COG1538) and Na^+^-driven multidrug efflux pump (COG0534), enriched in adults, and Transposase and inactivated derivatives (COG2801, COG2963), Transcriptional regulator/sugar kinase (COG1940) and Transcriptional regulator (COG1309), enriched in infants. All six COGs possess abundance profiles that are completely non-overlapping between infant and adult individuals (apart from COG1538, in which the lowest level in adults is slightly lower than the highest in infants) and are thus nominally quite discriminative. On the other hand, among the 192 COGs found by Metastats, 9 are not detected by LEfSe even at the lowest LDA score threshold (Additional file 6). All possess overlapping abundance values between infant and adult classes (at least two, and often more, of the highest samples in the less abundant class overlap the putatively more abundant class). This lack of discriminatory power precludes LEfSe from highlighting the differences as significant between adults and infants, particularly given the low number of infant samples.

Intriguingly, LEfSe's distinct list of functional activities in the core infant and adult microbiomes is suggestive of 'generalist' microbial activity during early life and specialization over time [76]. In fact, inspecting the five differentially abundant COGs with the highest effect sizes for each class, we find for infants very high-level functional groups related to broad transcriptional regulation (COG1609, COG1940, COG1309 and COG3711). In adults, all five represent more specialized orthologous groups, including COG1629 (Outer membrane receptor proteins, mostly Fe transport), COG1595 (DNA-directed RNA polymerase specialized sigma subunit, sigma24 homolog), and COG4771 (Outer membrane receptor for ferrienterochelin and colicins). Since the number of differentially abundant COGs is very high (366), this observation was only highlighted at the top of the candidate biomarker list due to LEfSe's effect size quantification, which allows the most characteristic differences among classes to emerge. For the same reason, we can easily confirm that sugar metabolism plays a crucial role in the infant gut and iron metabolism in adults, as already stated in [45,73]; the COGs with the highest LDA scores indeed possess sugar and glucose functional activities for infants and iron-related functionality for adults.

### LEfSe achieves a very low false positive rate in synthetic data

**Figure 5:**
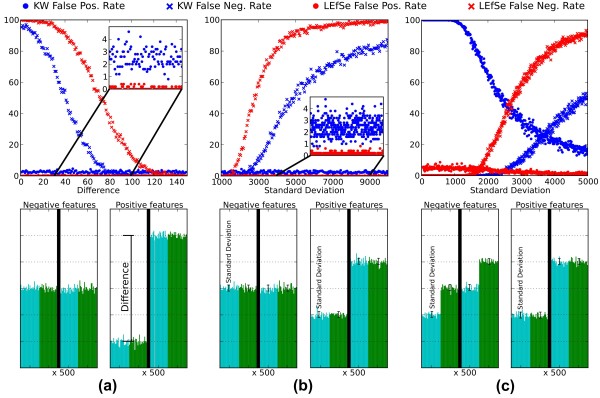
**Comparison of LEfSe and the KW test alone for false positive and negative rates in synthetic data**. Both tests used α = 0.05 in all cases, and the three artificial datasets comprise 100 samples, each in two classes, each with two subclasses of cardinality 25. The samples consist of 1,000 synthetic features taking the place of microbial taxa, pathways, and so on; half are negative (not biomarkers) and the other half positive. **(a) **LEfSe and KW false positive and negative rates at increasing values of the difference between class means. Negative features are normally distributed with parameters (μ = 10,000, σ = 100) across classes; positive features contain classes with increasingly different means. **(b) **Performance as standard deviation varies within classes (rather than the difference between means, fixed at 2,000). **(c) **Performance as standard deviation increases within inconsistent subclasses. Negative features have subclasses sampled from the same normal distribution (and thus not representing consistent biomarkers). Positive features are distributed as in (b). In all cases, LEfSe sacrifices a small number of false negatives in order to achieve a false positive rate near zero, with the goal of ensuring that biomarkers of large effect size will be both reproducible and biologically interpretable.

We further investigated the ability of LEfSe to detect biomarkers using synthetic high-dimensional data (see Materials and methods for the description of the dataset) in comparison with the KW test alone (a non-parametric adaptation of the analysis of variance (ANOVA)) and with Metastats [45]. The LDA effect size step of LEfSe is not considered here for simplicity, and the artificial data are detailed in Figure 5.

Theoretically, the settings of the first two experiments (Figure 5a,b) exactly match the application conditions for the KW test. The false positive rate (mean 2.5%, regardless of the distance between feature means and of the standard deviation of the normal distribution) is in fact consistent with the α value of 0.05, given that the negative features are half of the total. LEfSe behaved qualitatively very similar to KW, but with a considerably lower false positive rate (less than 0.5% in the great majority of the cases against a mean value of 2.5%) and a higher false negative rate. In biology, false positives are often perceived as more dramatic than false negatives [77-79]; this is often attributable to the fact that it is undesirable to invest in expensive experimental follow-up of false positives, whereas in high-throughput settings, a few true positives outweigh the false negatives that are left uninvestigated. With this motivation for minimizing false positives, we conclude that LEfSe performs at least as well as KW when no meaningful subclass structure is available. On the other hand, when subclasses can be identified internally to the classes and some of them do not agree with the trend among classes, LEfSe performs qualitatively and quantitatively much better than KW (Figure 5c). The false positives are in fact always substantially lower than KW, whereas the false negatives are higher only for very noisy features. Metastats [45] seems to achieve results very similar to KW (Additional file 7) with the same disadvantages with respect to LEfSe.

## Conclusions

Gaining insight into the structure, organization, and function of microbial communities has been proposed as one of the major research challenges of the current decade [80], and it will be enabled by both experimental and computational metagenomic analyses. To this end, we have developed the LEfSe algorithm for comparative metagenomic studies, permitting the characterization of microbial taxa specific to an experimental or environmental condition, the detection of pathways and biological mechanisms over- or under-represented in different communities, and the identification of metagenomic biomarkers in mammalian microbiomes. LEfSe is shown here to be effective in detecting differentially abundant features in the human microbiome (characteristically mucosal or aerobic taxa) and in a mouse model of colitis. A comparison with existing statistical methods and state-of-the-art metagenomic analyses of environmental, infant gut microbiome, and synthetic data shows that LEfSe consistently provides lower false positive rates and can effectively aid in explaining the biology underlying differences in microbial communities.

These findings demonstrate that a concept of class explanation including both statistical and biological significance is highly beneficial in tackling the statistical challenges associated with high-dimensional biomarker discovery [28,81,82]. Specifically, LEfSe determines features potentially able to explain the differences among conditions rather than the features that simply possess uneven distributions among classes. This is distinct from most current statistical approaches [45] and akin to the incorporation of biological prior knowledge that has proven highly successful in recent genome-wide association studies [83-85]. Moreover, particularly in (often noisy) metagenomic datasets, effect size can serve as an orthogonal measure to complement ranking biomarkers based on *P*-values alone. Differences between classes can be very statistically significant (low *P*-value) but so small that they are unlikely to be biologically responsible for phenotypic differences. On the other hand, a biomarker with a relatively large *P*-value (for example, 0.01) may correspond to a large effect size, with statistical significance diminished by technical noise. LEfSe investigates both aspects computationally by testing both the consistency and the effect size of differences in feature abundance among classes with respect to the structure of the problem. This is performed subsequently to standard statistical significance tests and is integrated in LEfSe by assessing biologically meaningful groups of samples among subclasses within each condition. This coupling of statistical approaches with biological consistency and effect size estimation alleviates possible artifacts or statistical inhomogeneity known to be common in metagenomic data, for example, extreme variability among subjects or the presence of a long tail of rare organisms [32,86]. Similarly, while multiple hypothesis corrected statistical significance speaks to the potential reproducibility of a result, estimation of effect size in high-dimensional settings is crucial for addressing biological consistency and interpretability.

The biology highlighted by these investigations speaks to the potential of metagenomics for both microbial ecology and translational applications. For example, certain bacterial clades are frequently detected as biomarkers even in diverse environments, suggesting that some species can adapt in surprisingly condition-specific manners. *Staphylococcus *and the Bacillales, for example, are discriminative for mucosal tissues, aerobic conditions, and murine colitis, whereas no Proteobacteria consistently characterize any of these conditions, even though they always represent a substantial portion of the communities. These observations may reflect extensive microenvironmental heterogeneity and the coexistence of generalist and specialist bacteria [87-89].

In addition to these insights into microbiology, metagenomic biomarkers, including the abundances of specific organisms, abundances of entire clades, or the presence/absence of specific organisms, can serve to describe host phenotypes, lifestyle, diet, and disease as well [5-10]. If the depletion of *Bifidobacterium *species in ulcerative colitis proves to occur early in human disease etiology, this and comparable shifts in the microbiota have potential applications in the detection of human disorders [90,91], especially as shifts in some bacterial consortia can be detected easily and inexpensively. Oral microbial biomarkers, for example, can be easily acquired and analyzed with microarray chips targeted for bacterial profiling [92]. These appear particularly promising for clinical applications [11], as the microbial communities in the saliva seem to represent one potential proxy for other human microbiota [93]. Other important clinical applications of metagenomic analyses include probiotic treatments [94,95] and microbiome transplantation [96-99] for gastrointestinal diseases.

LEfSe, the computational approach to biomarker class comparisons detailed here, thus contributes to the understanding of microbial communities and guides biologists in detecting novel metagenomic biomarkers. The algorithm's effectiveness on real and synthetic data has been highlighted by several experiments in which we successfully characterized both host-associated microbiota and environmental microbiomes in multiple contexts. To support ongoing metagenomic analyses, we have implemented LEfSe as a user-friendly web application that can provide both raw data and publication-ready graphical results, including reports of detected microbial variation on taxonomic trees for visual and biological summarization. LEfSe is freely available online in the Galaxy workflow framework [46,47] at the following link [48].

## Materials and methods

**Figure 6:**
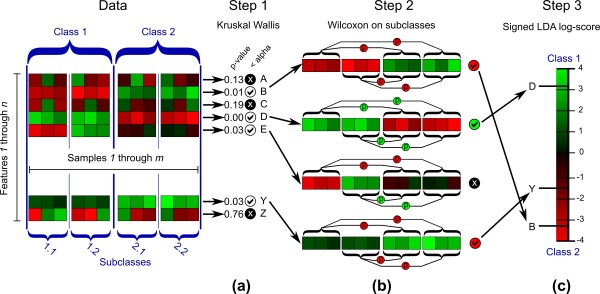
**Schematic representation of the statistical and computational steps implemented in LEfSe**. Input data consist of a collection of *m *samples (columns) each made up of *n *numerical features (rows, typically normalized per-sample, red representing high values and green low). These samples are labeled with a class (taking two or more possible values) that represents the main biological comparison under investigation; they may also have one or more subclass labels reflecting within-class groupings. **(a) **Step 1 analyzes all features, testing whether values in different classes are differentially distributed. **(b) **Features violating the null hypothesis are further analyzed in step 2, which tests whether all pairwise comparisons between subclasses in different classes significantly agree with the class level trend. **(c) **The resulting subset of vectors is used to build a LDA model from which the relative difference among classes is used to rank the features. The final output thus consists of a list of features that are discriminative with respect to the classes, consistent with the subclass grouping within classes, and ranked according to the effect size with which they differentiate classes.

The LEfSe algorithm is introduced in overview in the Results section, and Figure 6 illustrates in detail the format of the input (a matrix with *n *rows and *m *columns) and the three steps performed by the computational tool: the KW rank sum test [49] on classes, the pairwise Wilcoxon test [50,51] between subclasses of different classes, and the LDA [52] on the relevant features.

Each of the *n *features is represented with a positive-valued vector containing its abundances in the *m *samples, and each sample is associated with values describing its class and, optionally, subclass and/or originating subject. The factorial KW rank sum test is applied to each feature with respect to the class factor; the subclass and subject information are used as stratifying subgroups when present. Features that, according to the KW rank sum test, do not violate the null hypothesis of identical value distribution among classes (with default *P*-value, α = 0.05) are not further analyzed. The pairwise Wilcoxon test is applied to retained features belonging to subclasses of different classes. For each feature, the pairwise Wilcoxon test is not satisfied if at least one comparison between subclasses has a *P*-value higher than the chosen α or if the sign of variation is not equal among all comparisons. For example, if a feature appears in samples from two classes with three subclasses each, all nine comparisons between subclasses in different classes must violate the null hypothesis, and all signs of the differences between medians must be consistent. The features that pass the pairwise Wilcoxon test are considered successful biomarkers. An LDA model is finally built with the class as dependent variable and the remaining feature values, subclass, and subject values as independent variables. This model is used to estimate their effect sizes, which are obtained by averaging the differences between class means (using unmodified feature values) with the differences between class means along the first linear discriminant axis, which equally weights features' variability and discriminatory power. The LDA score for each biomarker is obtained computing the logarithm (base 10) of this value after being scaled in the [1,10^6^] interval and, regardless of the absolute values of the LDA score, it induces the ranking of biomarker relevance. For robustness, LDA is additionally supported by bootstrapping (default 30-fold) and subsequent averaging.

LEfSe's first two steps employ non-parametric tests because of the nature of metagenomic data. Relative abundances will, in most cases, violate the main assumption of typical parametric tests (normal population in each class), whereas non-parametric tests are much more robust to the underlying distribution of the data since they are distribution-free approaches. The only assumption of the Wilcoxon and KW tests is that the distributions in each class are identically shaped with possible differences in the medians. For example, the bimodal or multimodal abundance distribution of an organism violates the assumptions of parametric tests but not those of non-parametric tests, unless the number of peaks in the distribution (or, more generally, the shape of the distribution) also changes among classes. LDA is used for effect size estimation as our experiments determined it to more accurately estimate biological consistency compared to approaches like differences in group means/medians or support vector machines (SVMs) [100]. A comparison between LDA and SVM approaches for effect size estimation on the murine model of ulcerative colitis (for which low-throughput biological validations of biomarkers are available in [67]) is reported in our supplemental material (Additional files 8 and 9) and shows the advantages of LDA with respect to upranking features of potential biological interest. Theoretically, this is motivated by LDA's ability to find the axis of highest variance and SVM's focus on features' combined predictive power rather than single feature relevance. Note that as we are performing class comparison rather than class prediction, it is worth specifying that the effect size estimation accuracy of an algorithm is not directly connected with its predictive ability (for example, SVM approaches are generally considered more accurate than LDA for prediction).

### Multiclass strategies

Comparisons with more than two classes require special strategies for applying the Wilcoxon and LDA steps, whereas the factorial KW test is already appropriate for this setting. Our multiclass strategy for the Wilcoxon test depends on the problem-specific strategy chosen by the user to define features differentially distributed among the *n *classes. In the most stringent strategy, we require that all *n *abundance profiles of a feature are statistically significantly distinct among all *n *classes. This strategy, called 'strict', is implemented by requiring that all Wilcoxon tests between classes are significant. A more permissive strategy, called 'non-strict', considers a feature as a biomarker if at least one class is significantly different from all others. The more permissive strategy thus needs to satisfy only a subset of the Wilcoxon tests. Regardless of the strategy, the LDA step always reports the highest score detected among all pairwise class comparisons.

### Subclass structure variants encoding different biological hypotheses

Different interpretations of the biomarker class comparison problem are implemented in LEfSe by modifying the requirements for pairwise Wilcoxon comparisons among subclasses. If classes contain subclasses that represent distinct strata, we test only comparisons within each identical subclass (Figure 4). For example, to assess the effect of a treatment on two sub-types of the same disease, we compare pre- and post-treatment levels within each subclass and require that the trend observed at the class level is significant independently for both subclasses. To implement this variant, LEfSe performs the Wilcoxon step only comparing subclasses with the same name. Alternatively, subclasses may represent covariates within which feature levels may vary but for which the problem does not dictate explicit stratification (Figure 2). In both settings, we explicitly require all the pairwise comparison to reject the null hypothesis for detecting the biomarker; thus, no multiple testing corrections are needed.

### Subclasses containing few samples

When few samples are available, non-parametric tests like the Wilcoxon have reduced power to detect differences. This can affect LEfSe when subclasses are very small, preventing the overall test from even rejecting the null hypothesis. For this reason, small subclasses should be avoided when possible, for example, by excluding them from the problem or by grouping together all subclasses with small cardinalities. For cases in which removing or grouping subclasses is not possible or disrupts the biological consistency of the analysis, LEfSe substitutes the Wilcoxon test with a test to compare whether subclass medians differ with the expected sign. The user can choose the subclass cardinality threshold at which this median comparison is substituted for the Wilcoxon test.

### Parameter settings

Except as stated otherwise in Results, all experiments in this study were run with LEfSe's α parameter for pairwise tests set to 0.05 for both class normality and subclass tests, and the threshold on the logarithmic score of LDA analysis was set to 2.0. The stringency of these parameters is easily tunable (also through the web interface) and allows the user to detect biomarkers with lower *P*-values and/or higher effect size in order, for example, to prioritize additional biological experiments and validations. All LDA scores are determined by bootstrapping over 30 cycles, each sampling two-thirds of the data with replacement, with the maximum influence of the LDA coefficients in the LDA score of three orders of magnitude.

### Data description

Except as stated otherwise, taxonomic abundances for 16S samples were generated from filtered sequence reads using the RDP classifier [101], with confidences below 80% rebinned to 'uncertain'. For all the datasets described below, the final input for LEfSe is a matrix of relative abundances obtained from the read counts with per-sample normalization to sum to one. Witten-Bell smoothing [102] was used to accommodate rare types, but due to LEfSe's non-parametric approach, this has minimal effect on the discovered biomarkers and on the LDA score. This also allows our biomarker discovery method to avoid most effects of sequence quality issues as long as any sequencing biases are homogeneous among different conditions, as no specific assumptions on the statistical distribution and noise model are made by the algorithm as is standard for non-parametric approaches.

### Human microbiome data

The 16S rRNA-based phylometagenomic dataset of the normal (healthy) human microbiome was made available through the Human Microbiome Project [13], and consists of 454 FLX Titanium sequences spanning the V3 to V5 variable regions obtained for 301 samples from 24 healthy subjects (12 male, 12 female) enrolled at a single clinical site in Houston, TX. These samples cover 18 different body sites, including 6 main body site categories: the oral cavity (9 samples), the gut (1 sample), the vagina (3 samples), the retroauricular crease (2 samples), the nasal cavity (1 sample) and the skin (2 samples). Detailed protocols used for enrollment, sampling, DNA extraction, 16S amplification and sequencing are available on the Human Microbiome Project Data Analysis and Coordination Center website [103], and are also described elsewhere [55,56]. In brief, genomic DNA was isolated using the Mo Bio PowerSoil kit [104] and subjected to 16S amplifications using primers designed incorporating the FLX Titanium adapters and a sample barcode sequence, allowing directional sequencing covering variable regions V5 to partial V3 (primers: 357F 5'-CCTACGGGAGGCAGCAG-3' and 926R 5'-CCGTCAATTCMTTTRAGT-3'). Resulting sequences were processed using a data curation pipeline implemented in mothur [41], which reduces the sequencing error rate to less than 0.06% as validated on a mock community. As part of the pipeline parameters, to pass the initial quality control step, one unambiguous mismatch to the sample barcode and two mismatches to the PCR amplification primers were allowed. Sequences with an ambiguous base call or a homopolymer longer than eight nucleotides were removed from subsequent analyses, as suggested previously [105]. Based on the supplied quality scores, all sequences were trimmed when a base call with a score below 20 was encountered. All sequences were aligned using a NAST-based sequence aligner to a custom reference based on the SILVA alignment [106,107]. Sequences that were shorter than 200 bp or that did not align to the anticipated region of the reference alignment were removed from further analysis. Chimeric sequences were identified using the mothur implementation of the ChimeraSlayer algorithm [108]. Unique reads were classified with the MSU RDP classifier v2.2 [58] using the taxonomy proposed by [109], maintained at the RDP (RDP 10 database, version 6). The 16S rRNA reads are available in the Sequence Read Archive at [110].

### *T-bet^-/- ^× Rag2^-/- ^*and *Rag2^-/- ^*mouse data

*T-bet*^-/- ^× *Rag2*^-/- ^and *Rag2*^-/- ^mice, their husbandry, and their chow have been described in [67]. The animal studies and experiments were approved and carried out according to Harvard University's Standing Committee on Animals as well as National Institutes of Health guidelines. Collection, processing, and extraction of DNA from fecal samples were performed as described in [67]. The V5 and V6 regions of the 16S rRNA gene were targeted for amplification and multiplex pyrosequencing with error-correcting barcodes. Sequencing was performed using a Roche FLX Genome Sequencer at DNAVision (Charleroi, Belgium) and data were preprocessed to remove sequences with low-quality scores. There were 7,579 ± 2,379 high-quality 16S reads per sample with a mean read length of 278 bp.

### Viral and microbial environmental data

We retrieved from the online supplemental material of [69] the 80 available metagenomes (42 viromes, 38 microbiomes). We identified three environments containing at least seven samples and grouped them into coral, hyper-saline, and marine subclasses; the fourth subclass, other, groups all environments with few samples.

### Infant and adult microbiome data

The COG profiles of the nine adult and four unweaned infant microbiomes were obtained from the supplemental material of [73] and used unmodified in this study.

### Synthetic datasets

We built three collections of artificial datasets in order to compare LEfSe to KW and Metastats. All datasets have 1,000 features and 100 samples belonging evenly to two classes, and the values are sampled from a Gaussian normal distribution. The samples in the two classes are further organized in four subclasses (two per class) with equal cardinality. Of the 1,000 features, 500 features have different means across classes and should thus be detected as biomarkers (positive features), the other 500 features are evenly distributed among classes or among at least one subclass in both classes and should not be detected as discriminative (negative features). The methods are evaluated assessing the false positive rate (number of erroneously detected biomarkers with respect to the total number of features) and the false negative rate (number of correctly detected non-discriminant features with respect to the total number of features, that is, sensitivity). The three collections of datasets (graphically shown in Figure 5) differ in the distribution of values in the subclasses and in the mean/standard deviation of the normal distribution. (a) The subclasses in the same class have the same parameters (thus the subclass organization is meaningless). Negative features all have μ = 10,000 and σ = 100, whereas one class of the positive features has μ = 10,000 - t (σ = 100) and the other μ = 10,000 + t (σ = 100) where t is a parameter ranging from 1 to 150. The performances of all methods are assessed at regular steps of the t parameter. (b) Datasets in this collection are defined in the same way as collection (a) but with t = 1,000 for all datasets and σ ranging from 1,000 to 10,000. (c) The negative class in the third collection has different subclass distribution. In particular, the second subclass of the first class has the same mean of the first subclass of the second class. The other two subclasses have different means (μ = 10,000 - t and μ = 10,000 + t, t = 1,000), but the feature is not considered differential since the difference is not consistent between subclasses. The positive features are defined in the same way as dataset (b).

### Implementation and availability of the method

LEfSe is implemented in Python and makes use of R statistical functions in the coin [111] and MASS [112] libraries through the rpy2 library [113] and of the matplotlib [114] library for graphical output. LEfSe is provided with a graphical interface in the Galaxy framework [46,47], which allows the user to select parameters (the primary three stringency parameters, the multiclass setting, and other computational, statistical, and graphical preferences), to pipeline data between modules in a workflow framework, to generate publication-quality graphical outputs, and to combine these results with other statistical and metagenomic analyses. LEfSe is available at [48].

## Abbreviations

bp: base pair; KW: Kruskal-Wallis; LDA: linear discriminant analysis; LEfSe: linear discriminant analysis effect size; PCR: polymerase chain reaction; RDP: Ribosomal Database Project; SVM: support vector machines.

## Authors' contributions

NS and CH conceived the study; NS and LM implemented the methodology; NS: JI: LW: DG: WG: and CH analyzed the results; NS: JI: LW: DG: WG: and CH wrote the manuscript. All authors read and approved the manuscript in its final form.

## Supplementary Material

Additional file 1 **Supplementary Figure S6**. Histogram of within-subject β-diversity (community dissimilarity) between different mucosal (red) and non-mucosal (green) body sites. Click here for file

Additional file 2 **Supplementary Figure S1**. Cladogram representing the differences between viromes and microbiomes on the subsystem framework. Click here for file

Additional file 3 **Supplementary Figure S2**. Histogram of LDA logarithmic scores of biomarkers found by LEfSe comparing microbiomes and viromes within the subsystem framework. Click here for file

Additional file 4 **Supplementary Figure S3**. Histogram of LDA logarithmic scores of COG biomarkers found by LEfSe comparing adult and infant microbiomes. Click here for file

Additional file 5 **Supplementary Figure S4**. Functional features (COGs) that are discrimantive for the comparison between adult and infant microbiomes according to LEfSe but not detected by Metastats among the discriminant features with LDA score higher than 3. If we consider all the discriminant features without threhold on LDA score, LEfSe identifies 366 COGs in total, 185 of which are not discriminant for Metastats. Click here for file

Additional file 6 **Supplementary Figure S5**. Functional features (COGs) that are discrimantive for the comparison between adult and infant microbiomes according to Metastats but not detected by LEfSe. Even if median and variance suggest the differences to be discriminative, there are always some microbiomes (at least two) that are overlapping between classes. This is due to the stringent α-value (0.01) set for the KW test in LEfSe and to the fact that we use non-parametric statistics (differently from Metastats). Notice, however, that even using a low α-value LEfSe detects many more biomarkers than metastats (366 versus 192). Click here for file

Additional file 7 **Supplementary Figure S9**. Comparison between LEfSe and Metastats using the synthetic data described in Figure 5 and in the Materials and methods. LEfSe was applied as detailed in the paper; for Metastats we used the default settings (that is, α = 0.05 and N_permutations _= 1,000) and, as for LEfSe and KW, we disabled the per-sample normalization as the features are independent. **(a,b) **Metastats has a higher false positive rate (average 5%) than LEfSe (average below 0.5%) and lower false negative rate. **(c) **When the subclass information is meaningful (see Figure 5 for the representation of the dataset), LEfSe performs substantially better than Metastats both in terms of false positive and false negatives. Overall, on these synthetic data, Metastats achieves very similar results compared to KW (Figure 5) and neither of them can make use of additional information regarding the within-class structure, thus achieving poor results compared to LEfSe when such kinds of information are available. Click here for file

Additional file 8 **Supplementary Figure S7**. SVM-based effect size estimation for the biomarkers found for the *Rag2^-/- ^*versus *T-bet^-/-^xRag2^-/- ^*comparison reported in Figure 3 of the manuscript. The LDA-based approach for assessing effect size (Figure 3) is closer to the biological follow-up experiments and is more visually consistent. The reason for LDA superiority over SVM approaches for effect size estimation is theoretically connected with the ability of LDA to find the axis with the highest variance, and the SVM effort on evaluating the combined feature predictive power rather than single feature relevance. It is worth specifying that the effect size estimation accuracy of an algorithm is not directly connected with its predictive ability (SVM approaches are usually considered more accurate than LDA for prediction). Click here for file

Additional file 9 **Supplementary Figure S8**. Comparison between the features with the highest SVM-based effect size (*Papillibacter*, on the left), the highest LDA-based effect size (*Bifidobacterium*, in the center), and the Actinobacteria phylum (on the right). From a visual analysis, *Bifidobacerium *shows a larger effect size, which is also evident looking at the ratios between class means, suggesting LDA as a better option for effect size estimation than SVM approaches. As detailed in the manuscript, the relevance of *Bifidobacterium *has been experimentally validated. Moreover, the large difference in the score given by the SVM approach to Actinobacteria compared to *Bifidobacterium *and *Papillibacter *is not consistent. Click here for file

Additional file 10 ***T-bet*^-/- ^× *Rag2*^-/- ^- *Rag2*^-/- ^dataset**. Input LEfSe file for the analysis of the ulcerative colitis phenotype in mice. Click here for file
