# Supplementary figures and images for: Metagenomic biomarker discovery and explanation
Source: Genome Biol. 2011 Jun 24;12(6):R60. doi: 10.1186/gb-2011-12-6-r60 (PMC3218848; doi:10.1186/gb-2011-12-6-r60)

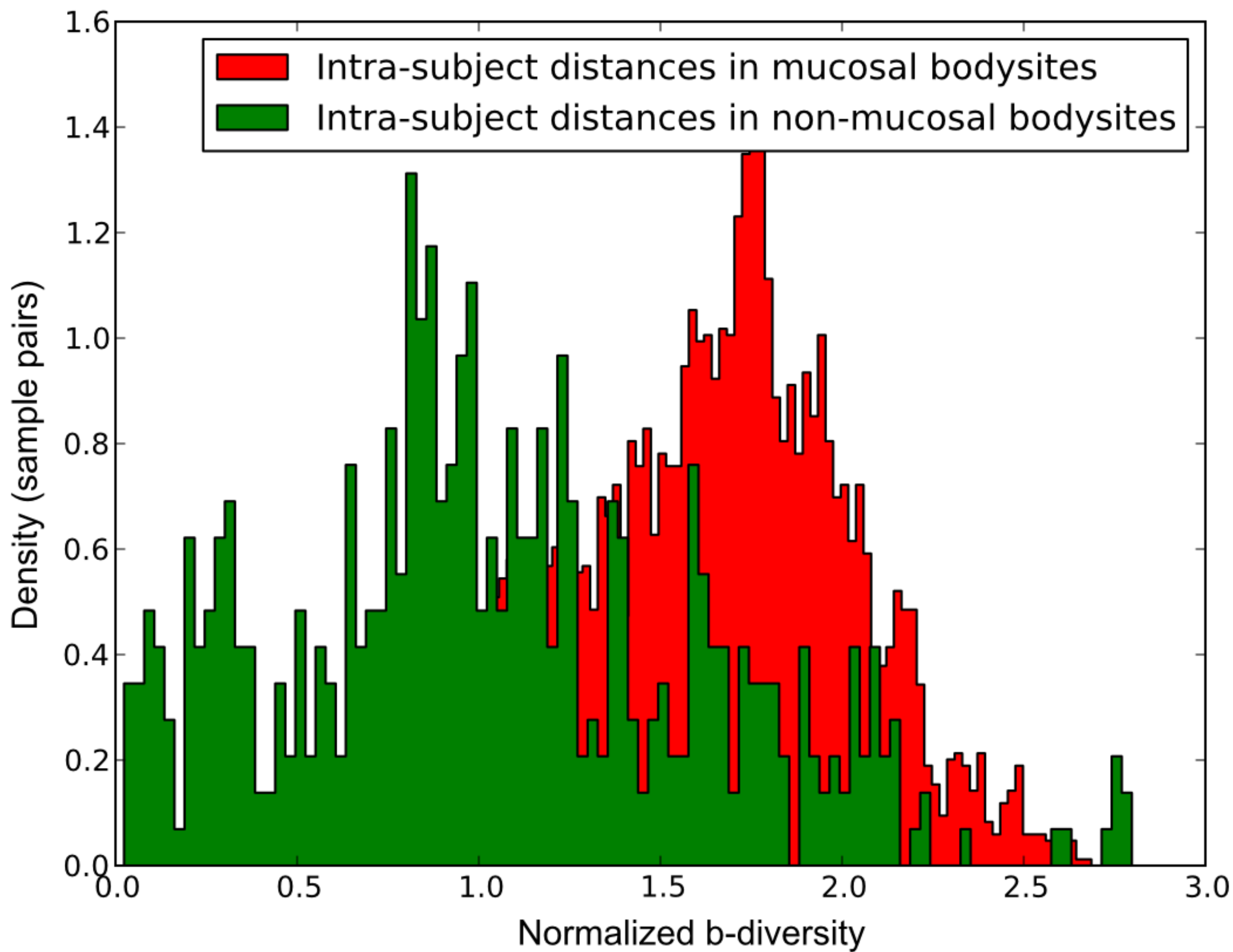

Supplement: Additional file 1 — Supplementary Figure S6. Histogram of within-subject β-diversity (community dissimilarity) between different mucosal (red) and non-mucosal (green) body sites. [file gb-2011-12-6-r60-S1.PDF]

**Microbiomes**

**Viromes**

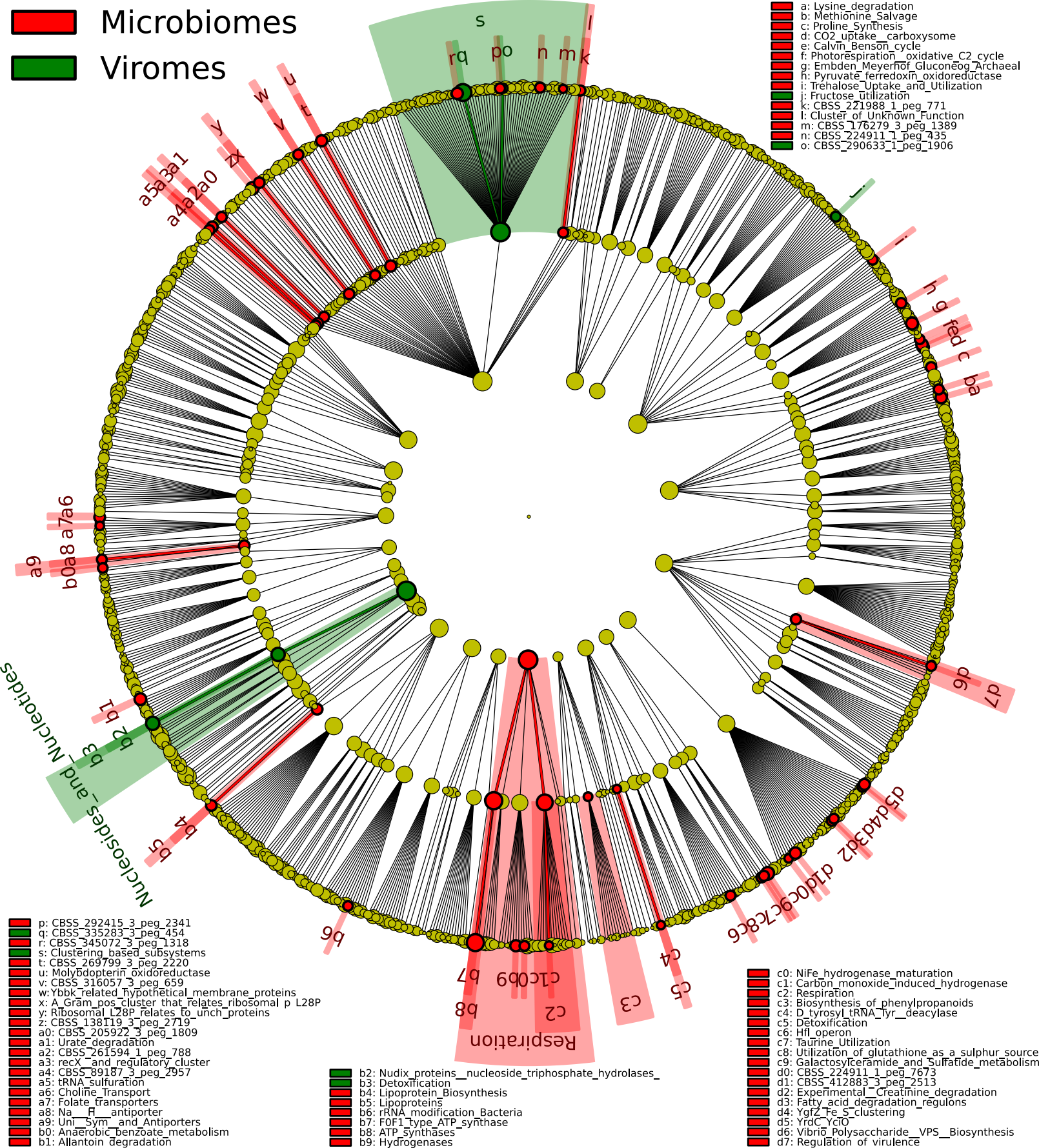

Supplement: Additional file 2 — Supplementary Figure S1. Cladogram representing the differences between viromes and microbiomes on the subsystem framework. [file gb-2011-12-6-r60-S2.PDF]

Microbiomes

Viromes

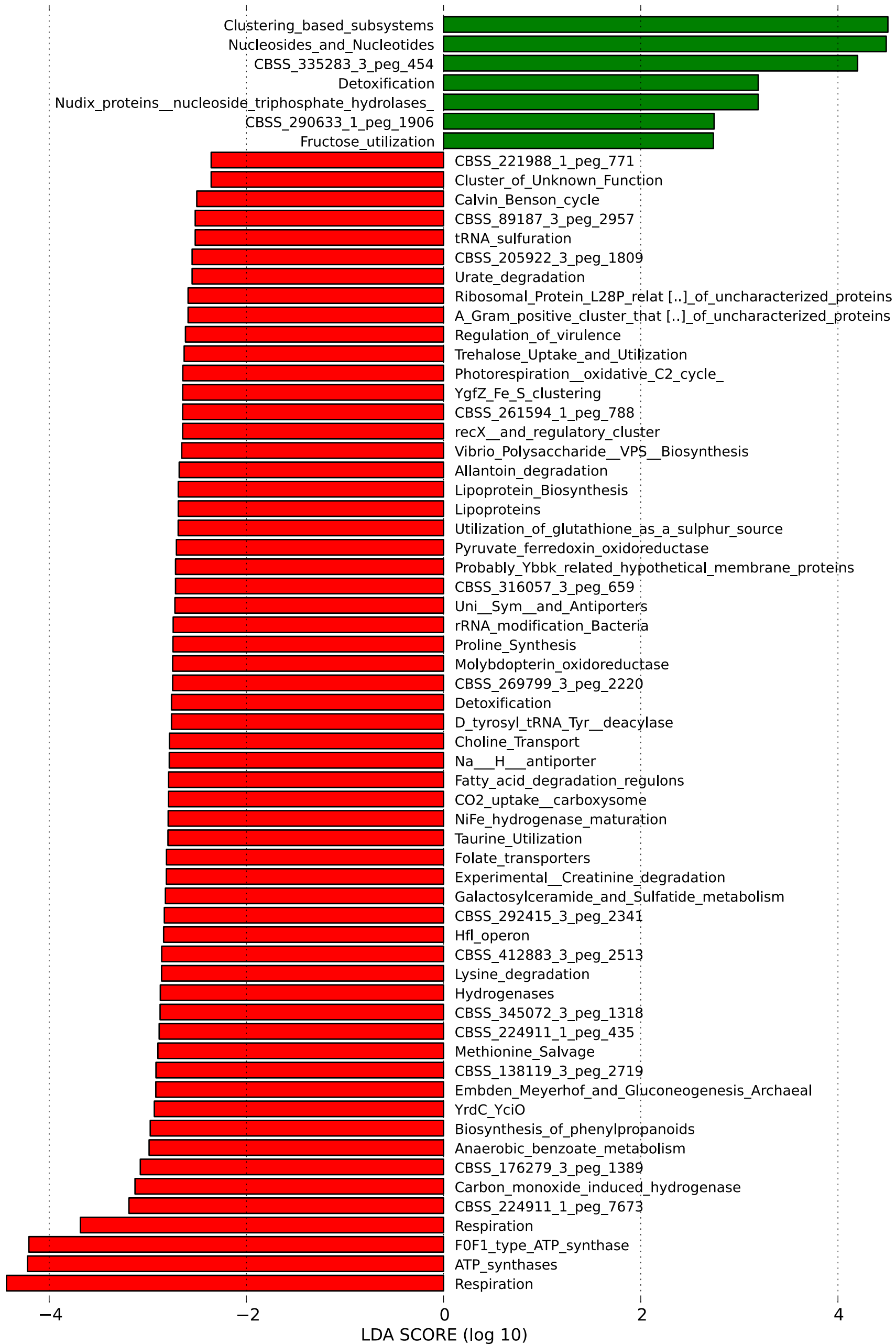

Supplement: Additional file 3 — Supplementary Figure S2. Histogram of LDA logarithmic scores of biomarkers found by LEfSe comparing microbiomes and viromes within the subsystem framework. [file gb-2011-12-6-r60-S3.PDF]

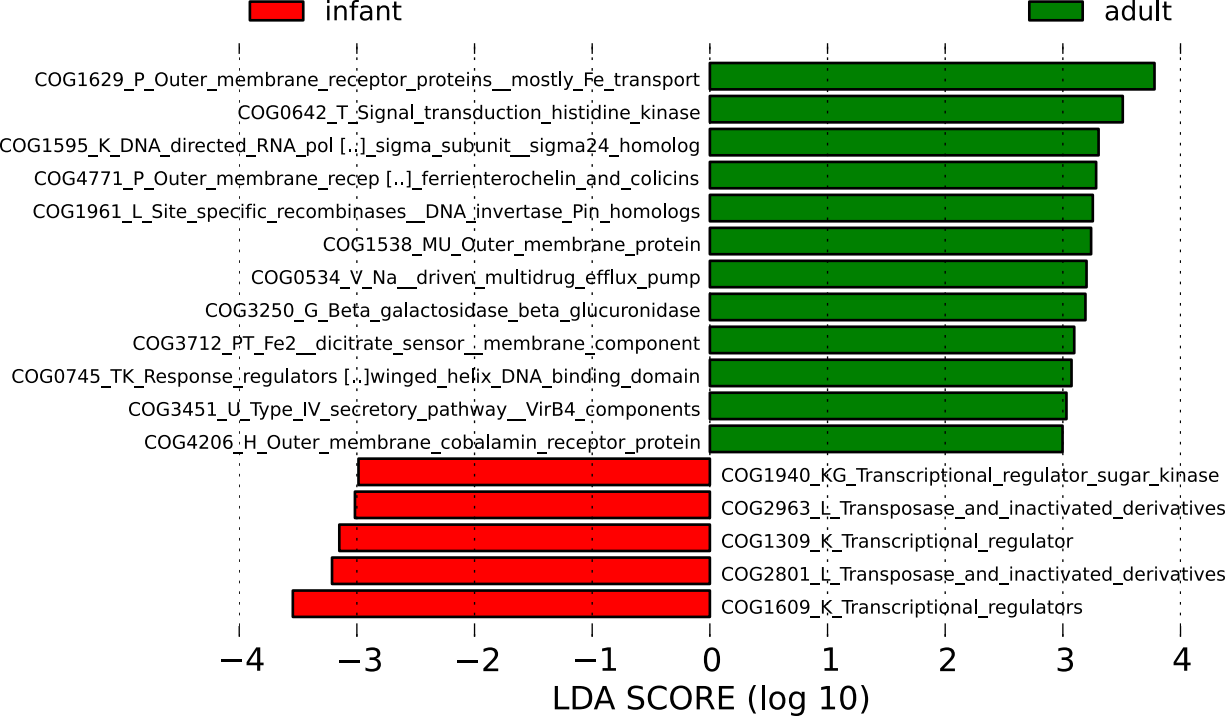

Supplement: Additional file 4 — Supplementary Figure S3. Histogram of LDA logarithmic scores of COG biomarkers found by LEfSe comparing adult and infant microbiomes. [file gb-2011-12-6-r60-S4.PDF]

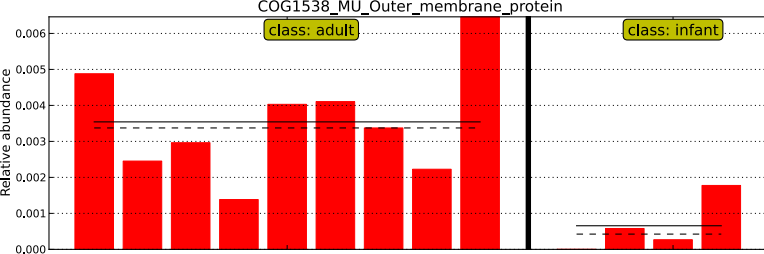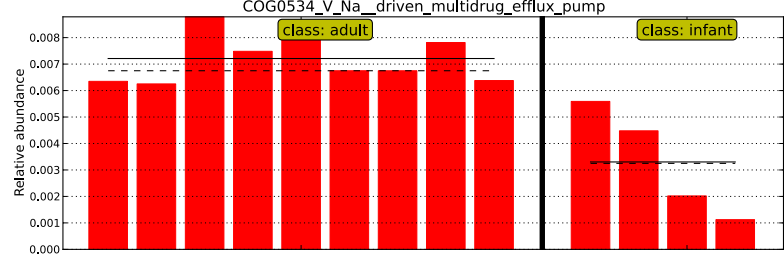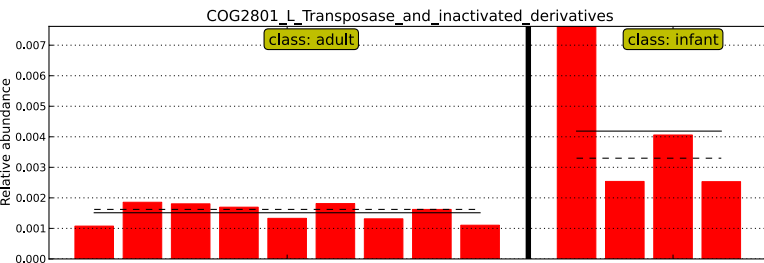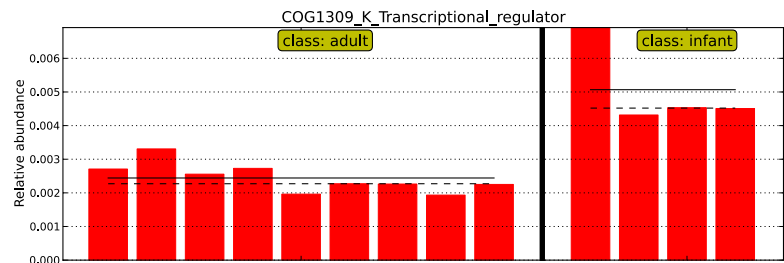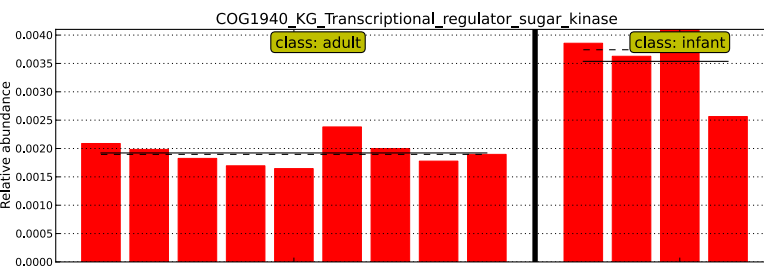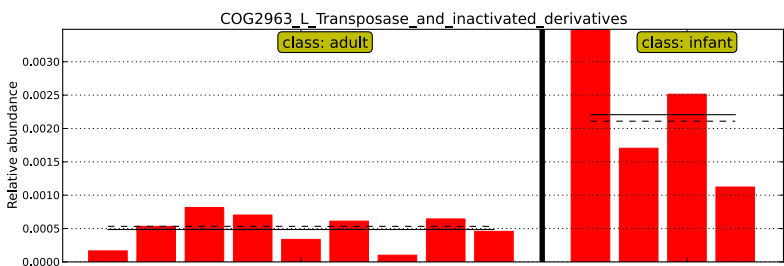

Supplement: Additional file 5 — Supplementary Figure S4. Functional features (COGs) that are discrimantive for the comparison between adult and infant microbiomes according to LEfSe but not detected by Metastats among the discriminant features with LDA score higher than 3. If we consider all the discriminant features without threhold on LDA score, LEfSe identifies 366 COGs in total, 185 of which are not discriminant for Metastats. [file gb-2011-12-6-r60-S5.PDF]

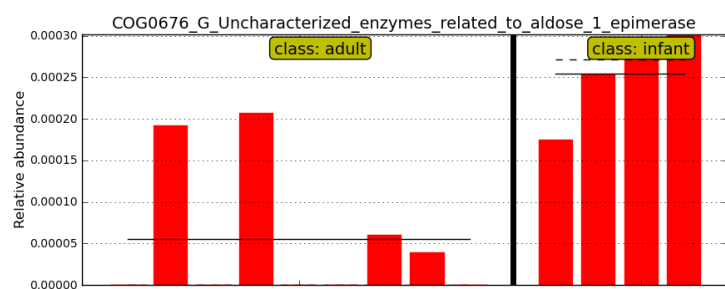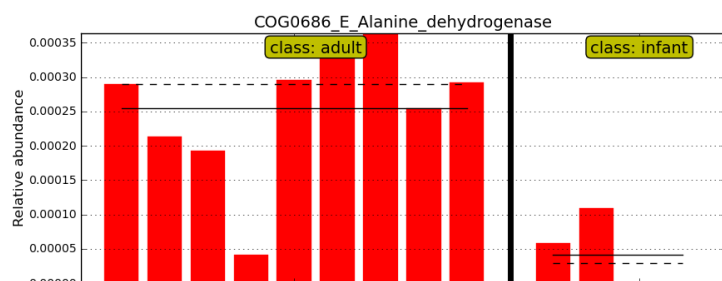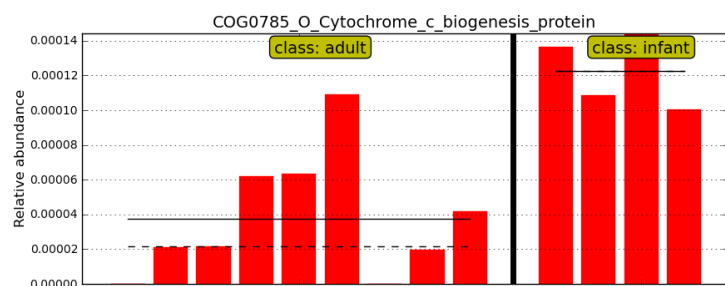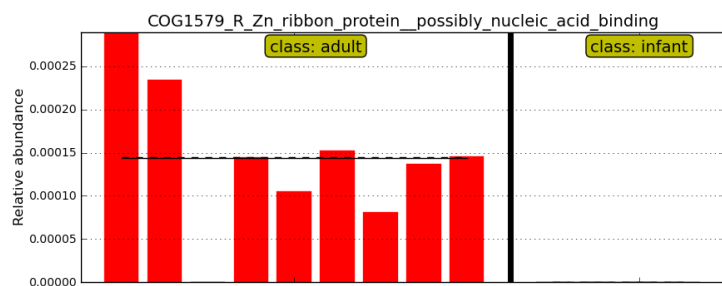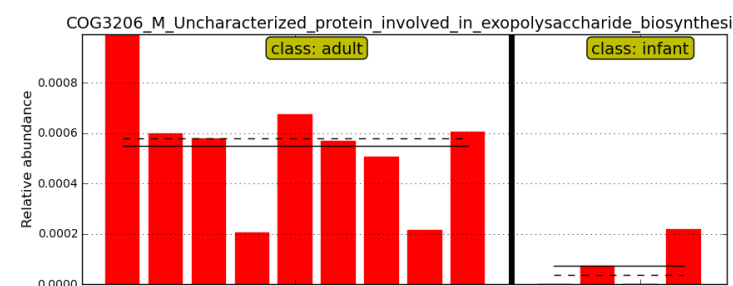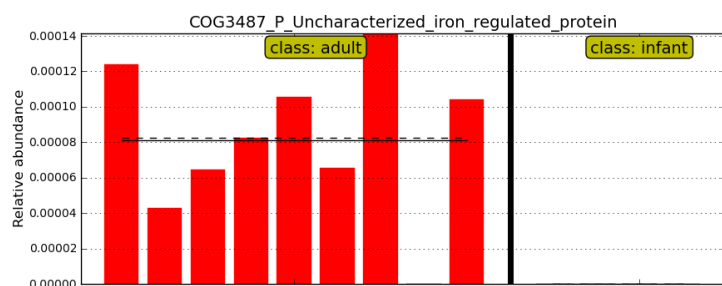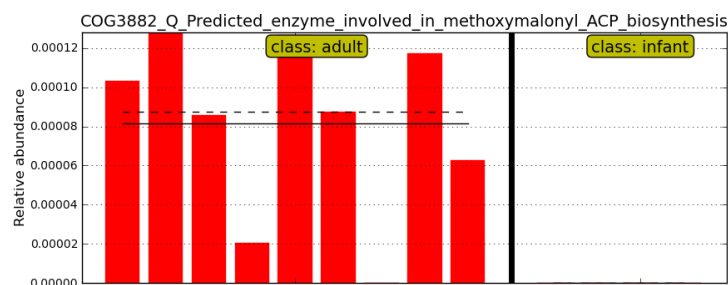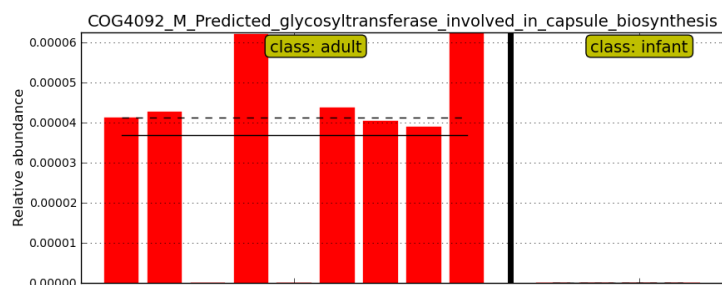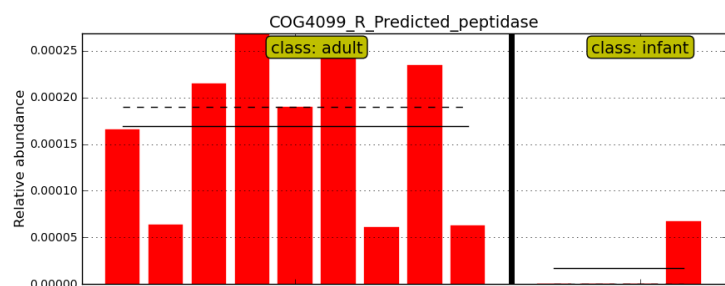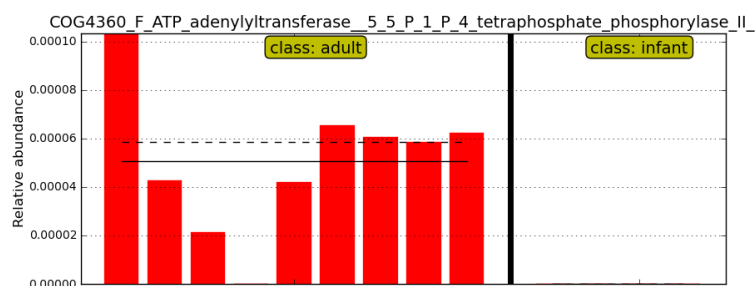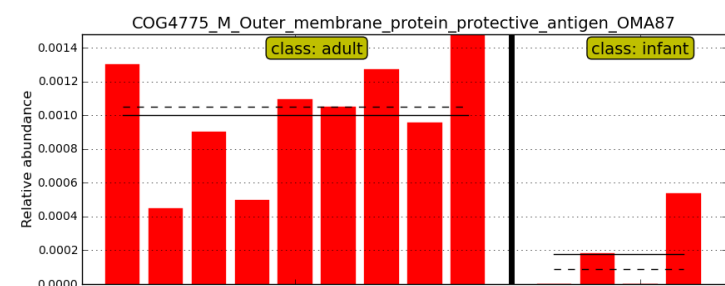

Supplement: Additional file 6 — Supplementary Figure S5. Functional features (COGs) that are discrimantive for the comparison between adult and infant microbiomes according to Metastats but not detected by LEfSe. Even if median and variance suggest the differences to be discriminative, there are always some microbiomes (at least two) that are overlapping between classes. This is due to the stringent α-value (0.01) set for the KW test in LEfSe and to the fact that we use non-parametric statistics (differently from Metastats). Notice, however, that even using a low α-value LEfSe detects many more biomarkers than metastats (366 versus 192). [file gb-2011-12-6-r60-S6.PDF]

● METASTATS False Pos. Rate    × METASTATS False Neg. Rate    ● LefSe False Pos. Rate    × LefSe False Neg. Rate

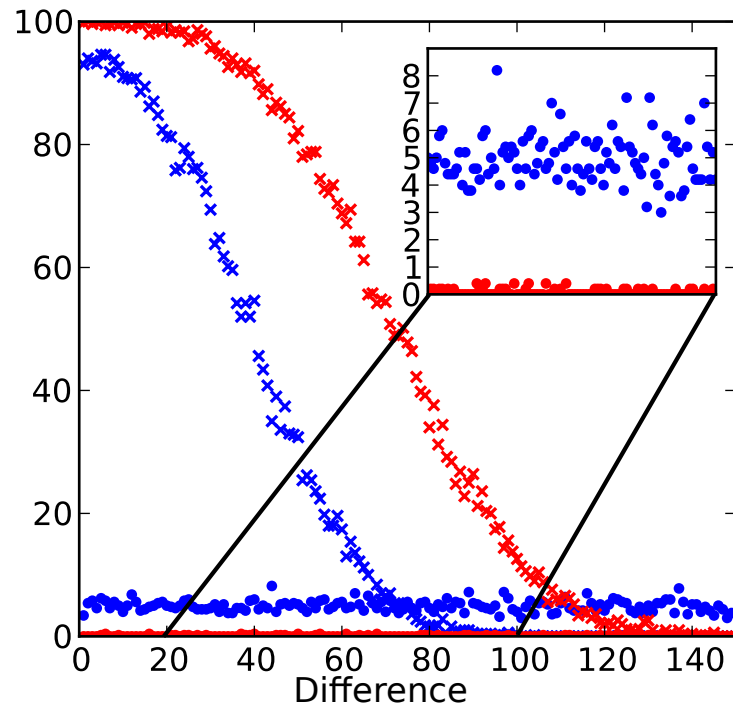

A

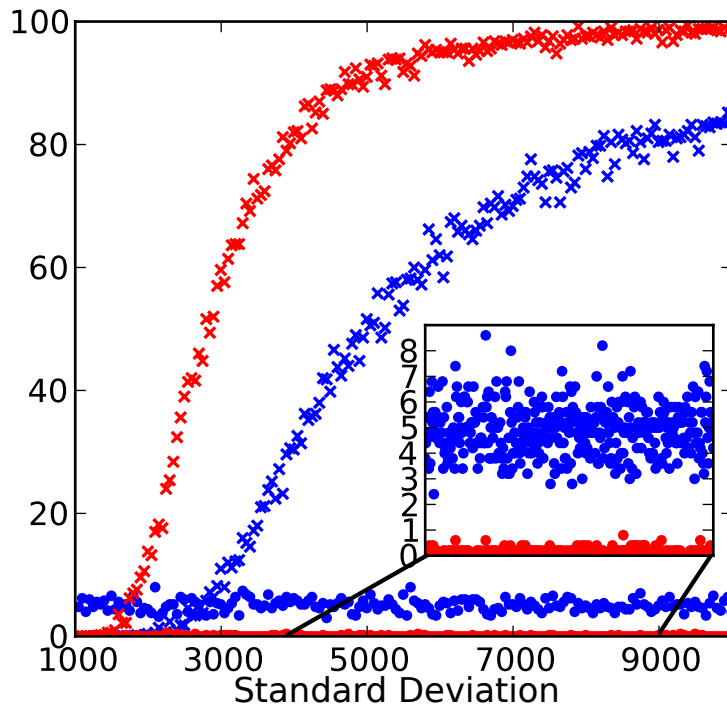

B

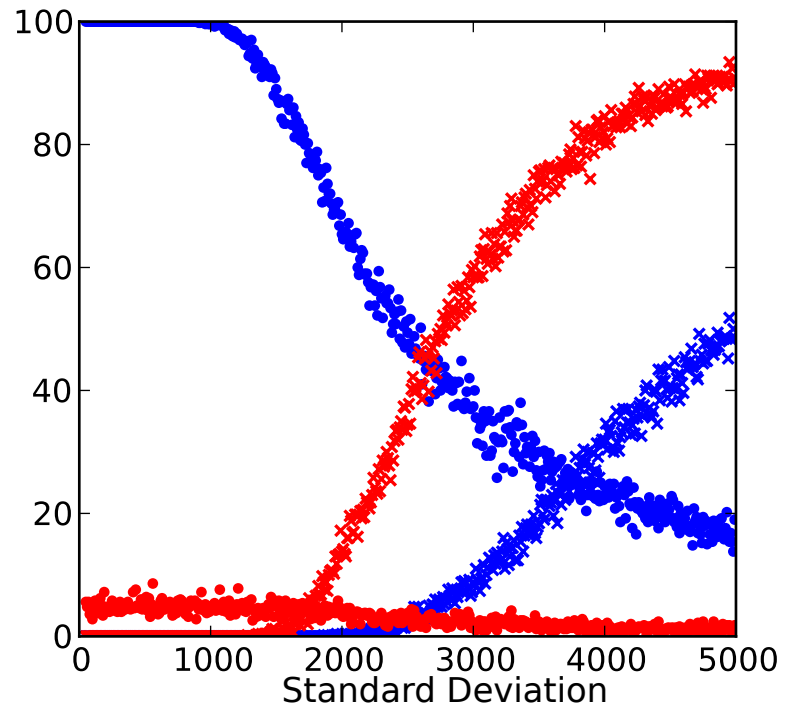

C

Supplement: Additional file 7 — Supplementary Figure S9. Comparison between LEfSe and Metastats using the synthetic data described in Figure 5 and in the Materials and methods. LEfSe was applied as detailed in the paper; for Metastats we used the default settings (that is, α = 0.05 and Npermutations = 1,000) and, as for LEfSe and KW, we disabled the per-sample normalization as the features are independent. (a,b) Metastats has a higher false positive rate (average 5%) than LEfSe (average below 0.5%) and lower false negative rate. (c) When the subclass information is meaningful (see Figure 5 for the representation of the dataset), LEfSe performs substantially better than Metastats both in terms of false positive and false negatives. Overall, on these synthetic data, Metastats achieves very similar results compared to KW (Figure 5) and neither of them can make use of additional information regarding the within-class structure, thus achieving poor results compared to LEfSe when such kinds of information are available. [file gb-2011-12-6-r60-S7.PDF]

Rag2<sup>-/-</sup>

T-bet<sup>-/-</sup>xRag2<sup>-/-</sup>

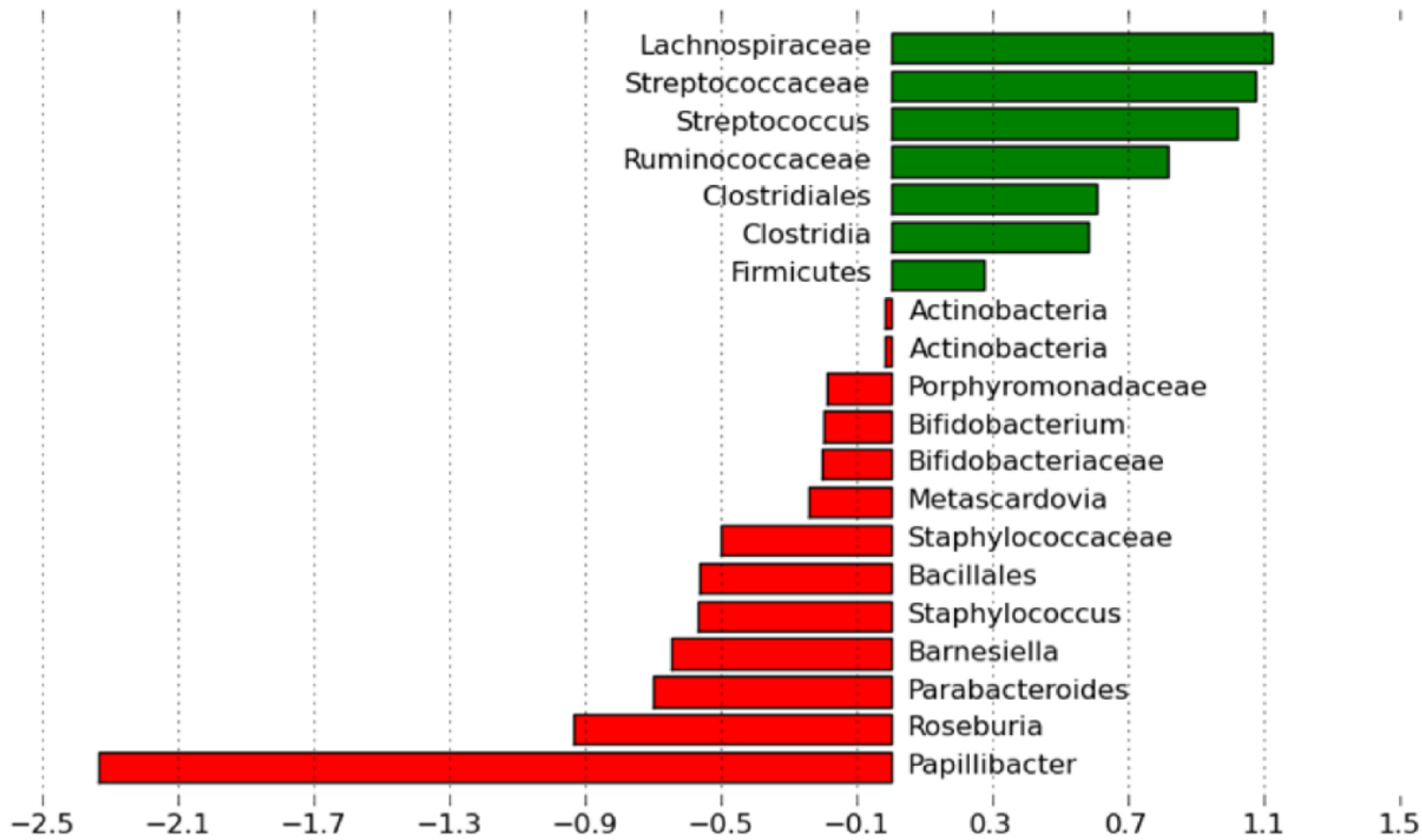

Supplement: Additional file 8 — Supplementary Figure S7. SVM-based effect size estimation for the biomarkers found for the Rag2-/- versus T-bet-/-xRag2-/- comparison reported in Figure 3 of the manuscript. The LDA-based approach for assessing effect size (Figure 3) is closer to the biological follow-up experiments and is more visually consistent. The reason for LDA superiority over SVM approaches for effect size estimation is theoretically connected with the ability of LDA to find the axis with the highest variance, and the SVM effort on evaluating the combined feature predictive power rather than single feature relevance. It is worth specifying that the effect size estimation accuracy of an algorithm is not directly connected with its predictive ability (SVM approaches are usually considered more accurate than LDA for prediction). [file gb-2011-12-6-r60-S8.PDF]
