# Supplementary material for: Metagenomic biomarker discovery and explanation
Source: Genome Biol. 2011 Jun 24;12(6):R60. doi: 10.1186/gb-2011-12-6-r60 (PMC3218848; doi:10.1186/gb-2011-12-6-r60)
Supplement: Additional file 9 — Supplementary Figure S8. Comparison between the features with the highest SVM-based effect size (Papillibacter, on the left), the highest LDA-based effect size (Bifidobacterium, in the center), and the Actinobacteria phylum (on the right). From a visual analysis, Bifidobacerium shows a larger effect size, which is also evident looking at the ratios between class means, suggesting LDA as a better option for effect size estimation than SVM approaches. As detailed in the manuscript, the relevance of Bifidobacterium has been experimentally validated. Moreover, the large difference in the score given by the SVM approach to Actinobacteria compared to Bifidobacterium and Papillibacter is not consistent. [file gb-2011-12-6-r60-S9.PDF]

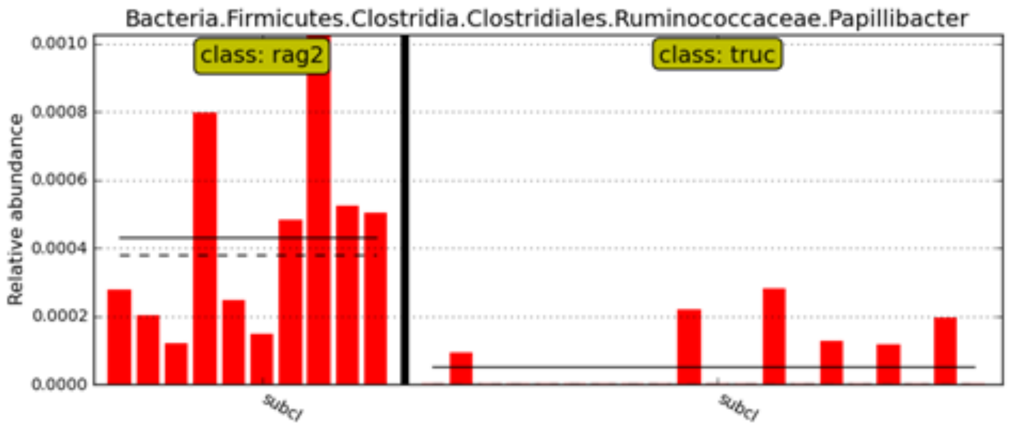
Bacteria.Firmicutes.Clostridia.Clostridiales.Ruminococcaceae.Papillibacter
class: rag2
class: truc
Relative abundance
0.0010
0.0008
0.0006
0.0004
0.0002
0.0000
subcl
subcl

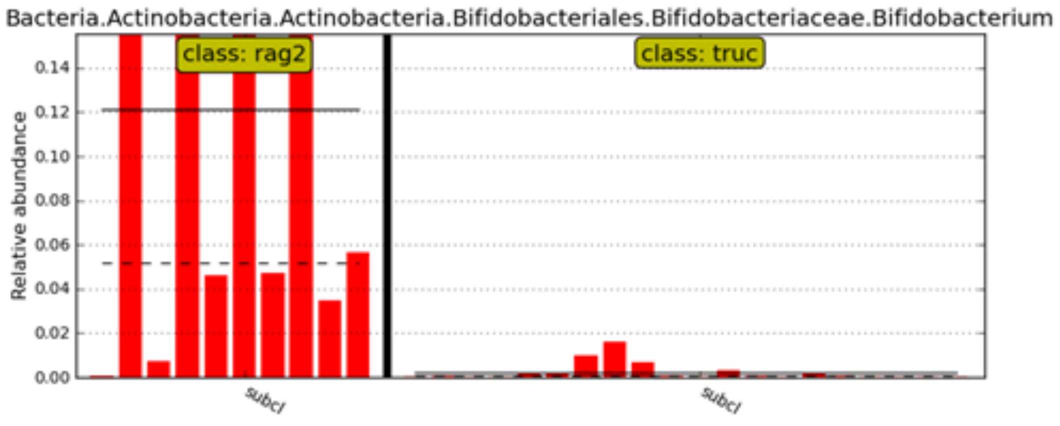
Bacteria.Actinobacteria.Actinobacteria.Bifidobacteriales.Bifidobacteriaceae.Bifidobacterium
class: rag2
class: truc
Relative abundance
0.14
0.12
0.10
0.08
0.06
0.04
0.02
0.00
subcl
subcl

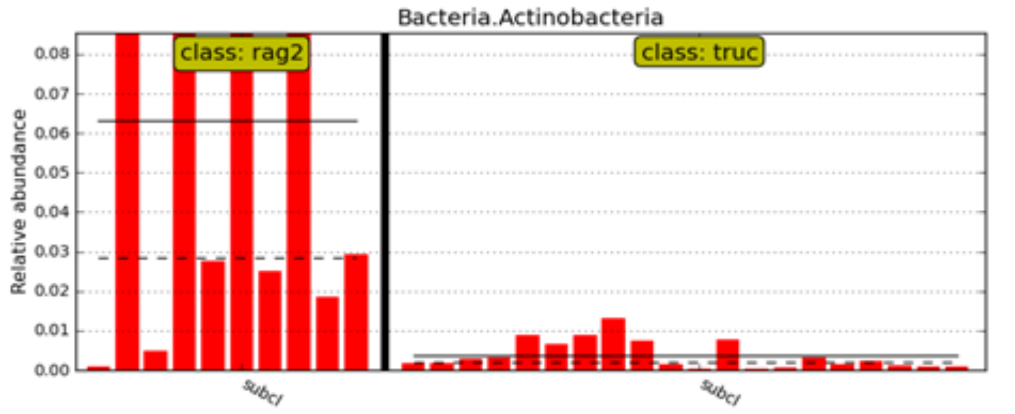
Bacteria.Actinobacteria
class: rag2
class: truc
Relative abundance
0.08
0.07
0.06
0.05
0.04
0.03
0.02
0.01
0.00
subcl
subcl
